# Early diagnosis and appropriate respiratory support for *Mycoplasma pneumoniae* pneumonia associated acute respiratory distress syndrome in young and adult patients: a case series from two centers

**DOI:** 10.1186/s12879-020-05085-5

**Published:** 2020-05-24

**Authors:** Lin Ding, Yu Zhao, Xuyan Li, Rui Wang, Ying Li, Xiao Tang, Bing Sun, Hangyong He

**Affiliations:** 1 Department of Respiratory and Critical Care Medicine, Beijing LuHe Hospital, Capital Medical University, Beijing, 101149 China; 2 Department of Respiratory and Critical Care Medicine, Beijing Institute of Respiratory Medicine, Beijing Key Laboratory of Respiratory and Pulmonary Circulation, Beijing Chao-Yang Hospital, Capital Medical University, No. 8 Gongren Tiyuchang Nanlu, Chaoyang District, Beijing, 100020 China

**Keywords:** *Mycoplasma pneumoniae*(*M. pneumoniae*), Acute respiratory distress syndrome (ARDS), Community-acquired pneumonia (CAP), High flow nasal cannula (HFNC), Extracorporeal membrane oxygenation (ECMO)

## Abstract

**Background:**

*Mycoplasma pneumoniae* (*M. pneumoniae*) is one of the most common causes of community acquired pneumonia (CAP). Establishing an early diagnosis of *M. pneumoniae* pneumonia in patients with acute respiratory distress syndrome (ARDS) may have important therapeutic implications.

**Methods:**

We describe diagnosis and management of *M. pneumoniae* pneumonia induced ARDS in a case series of adults and youth hospitalized with radiographically confirmed CAP prospectively enrolled in an observational cohort study in two university teaching hospitals, from November 2017 to October 2019.

**Results:**

In all 10 patients, early and rapid diagnosis for severe *M. pneumoniae* pneumonia with ARDS was achieved with polymerase chain reaction (PCR) or metagenomic next-generation sequencing (mNGS) testing of samples from the lower respiratory tract or pleural effusion. The average PaO_2_/FiO_2_ of all patients was 180 mmHg. Of the 10 cases, 4 cases had moderate ARDS (100 mmHg ≤ PaO_2_/FiO_2_ < 200 mmHg) and 3 cases had severe ARDS (PaO_2_/FiO_2_ < 100 mmHg). High flow nasal cannula (HFNC) was applied in all patients, though only two patients were sufficiently supported with HFNC. Invasive mechanical ventilation (IMV) was required in 5 patients. High resistance (median 15 L/cmH_2_O/s) and low compliance (median 38 ml/cmH_2_O) was observed in 4 cases. In these 4 cases, recruitment maneuvers (RM) were applied, with 1 patient demonstrating no response to RM. Prone positioning were applied in 4 cases. Two cases needed ECMO support with median support duration of 5.5 days. No patient in our case series received corticosteroid therapy. All patients were survived and were discharged from hospital.

**Conclusions:**

Early and rapid diagnosis of severe *M. pneumoniae* pneumonia with ARDS can be achieved with PCR/mNGS tests in samples from the lower respiratory tract or pleural effusion. In our case series, half of *M. pneumoniae* pneumonia induced ARDS cases were adequately supported with HFNC or NIV, while half of cases required intubation. RM and prone position were effective in 30% of intubated cases, and 20% needed ECMO support. When early anti-mycoplasmal antibiotics were given together with sufficient respiratory support, the survival rate was high with no need for corticosteroid use.

## Background

*Mycoplasma pneumoniae* (*M. pneumoniae*) is one of the most common causes of community acquired pneumonia (CAP) often seen in children and young adults, and accounts for 10–39% of all cases of adult CAP cases [1, 2]. *M. pneumoniae* pneumonia is typically mild and characterized by a persistent dry cough or self-limiting pneumonia that resolves with no medication [3]. However, respiratory failure and severe acute respiratory distress syndrome (ARDS) occur in 0.5–2% of all *M. pneumoniae* pneumonia cases and primarily affect young adults [4–18]. The rates of intensive care unit (ICU) admission of hospitalized *M. pneumoniae* pneumonia patients are reported as 10% in the US and 16.3% in Europe [19, 20]. The rate of ICU admission is even higher at 38.8% in patients older than 65 years, compared to 18% in patients older than 19 years [20]. In one retrospective study from our hospital, 4.1% of *M. pneumoniae* pneumonia patients needed ICU admission for acute respiratory failure in the setting of an epidemic [21].

Severe ARDS and fatal outcome as a result of *M. pneumoniae* pneumonia may be the result of unclear clinical features [5], delayed diagnosis, inappropriate respiratory support, and/or insufficient initial treatment. When acute nonbacterial pneumonia progresses, *M. pneumoniae* must be considered as a possible cause, and appropriate diagnosis, respiratory support and therapeutic measures should be promptly instituted. Previous studies suggest that *M. pneumoniae* infection should be included in the differential diagnosis of ARDS, and that establishing an early diagnosis may have important therapeutic implications [22].

In recent years, rapid diagnostic methods have been developed, allowing for early diagnosis of *M. pneumoniae* pneumonia. Detection of *M. pneumoniae* using fluorescence-quantatitive PCR in respiratory samples [19, 21–24] and metagenomic next-generation sequencing (mNGS) has increased [25]; these methods are especially useful for early detection of rare, atypical, and slow-growing microbes. Case reports have also described using new forms of respiratory support for *M. pneumoniae* pneumonia induced ARDS, such as high-flow nasal cannula (HFNC) [26], non-invasive ventilation (NIV) [27] and veno-venous extracorporeal membrane oxygenation (ECMO) [18, 24, 28]. However, there has not yet been a full evaluation of the new available diagnostic and therapeutic measures in *M. pneumoniae* pneumonia induced ARDS.

The aim of our study was to describe a case series of 10 patients with *M. pneumoniae* pneumonia induced ARDS and provide an overview of available modalities for diagnosis and treatment. We describe the epidemiological, clinical, imaging, and laboratory features of our patients, review the available procedures for early diagnosis, and evaluate available respiratory support techniques in clinical practice in order to highlight the importance of rapid recognition and appropriate treatment.

## Methods

### Study population

We retrospectively identified all cases of young and adult patients (age over 14 years) with ARDS caused by *M. pneumoniae* pneumonia who were admitted to the respiratory ICU in two teaching hospitals (Beijing Chao-Yang Hospital and Beijing Luhe Hospital, Beijing, China) with radiographically confirmed CAP from November 2017 to October 2019. The study was approved by the institutional review boards (IRB) at each institution. Written informed consent was obtained from all participants, where participants are children (under 16 years old) from their parent or guardian.

### Case definitions for *M. pneumoniae* pneumonia induced ARDS

Criteria for diagnosis of *M. pneumoniae* pneumonia were based on 1) clinical signs and symptoms (cough, fever, productive sputum, dyspnoea, chest pain or abnormal breath sounds); 2) radiographic pulmonary abnormalities that were at least segmental and were not explained by pre-existing or other known causes; and 3) positive detection of *M. pneumoniae* nucleic acid by PCR or mNGS from lower respiratory tract secretion (sputum and BALF), which were considered as microbiological evidence of infection.

All cases of pneumonia occurring more than 3 days after hospitalization were considered nosocomial and were excluded. Patients with HIV infection, neutropenia, or who were receiving immunosuppressive chemotherapy were excluded.

The diagnosis of ARDS was assigned to patients who met the Berlin definition criteria: 1) presence of acute hypoxemic respiratory failure; 2) onset within 7 days of insult, or new (within 7 days) or worsening respiratory symptoms; 3) bilateral opacities on chest x-ray or CT not fully explained by effusions, lobar or lung collapse, or nodules; and 4) cardiac failure not the primary cause of acute respiratory failure. We categorized patients into 3 mutually exclusive classes of ARDS severity using previous definitions based on degree of hypoxemia: 1) mild (200 mmHg ≤ PaO_2_/FIO_2_ < 300 mmHg); 2) moderate (100 mmHg ≤ PaO_2_/FIO_2_ < 200 mmHg); and 3) severe (PaO_2_/FIO_2_ < 100 mmHg).

### Microbiological tests

Patient specimens, including sputum, whole blood, and serum samples, were collected upon admission and during hospitalization for bacterial and viral testing. Microbiological tests were performed at the Department of Infectious Disease and Clinical Microbiology laboratories in our centers. Throat swabs, sputum, endotracheal aspiration or BALF were collected for *M. pneumoniae* PCR assay. All severe ARDS patients had *M. pneumoniae* infection confirmed by PCR assay or mNGS from lower respiratory tract secretion (sputum and BALF). PCR of pleural effusion fluid were also tested in some patients for the detection of *M. pneumoniae*.

### Data collection

Clinical information collected included the following: characteristics (age and sex), comorbidities, clinical symptoms (fever, cough, sputum, dyspnea, chest pain, rash, nausea, vomiting, abdominal pain, diarrhea and headache), clinical signs (body temperature, heart rate, respiratory frequency, blood pressure and crackles in the lungs), laboratory tests (whole-blood cell count and blood chemistry), and microbiological findings and images of the lung, including chest X-ray (CXR) and high resolution computed tomography (HRCT). Concomitant medications, respiratory support (HFNC, NIV, invasive mechanical ventilation, prone position and ECMO), complications, and outcomes were also recorded.

### Statistical analysis

Pooled epidemiological, clinical, imaging, and laboratory data are shown as median with range for quantitative variables and as absolute and relative frequencies for qualitative variables. The enrolled patients were divided into two groups based on use of invasive mechanical ventilation. Continuous variables were compared using the Mann-Whitney U-test, whereas categorical data were compared using the Chi-squared test or the Fisher’s Exact test, where appropriate. All comparisons were performed using the SPSS statistics package version 19.0. Differences were considered statistically significant when *p* was < 0.05.

## Results

### Baseline characteristics of the included patients

Between November 2017 and October 2019, 11 patients met criteria of severe *M. pneumoniae* pneumonia. Of the 11 patients, one patient was excluded due to diagnosis of lymphoma combined with adenovirus pneumonia. Therefore, 10 immunocompetent patients were included in the final analysis.

The age range of our patients was 15 to 53 (median 31) years. There were 6 male and 4 female patients. All cases were admitted in different months of the year except for January, February and September. Only one patient (case 9) had diabetes mellitus. The other 9 patients had no underlying diseases (Table 1).

**Table 1:** Clinical characteristics and diagnostics for the patients with severe *M. pneumoniae* pneumonia

Case No.	Male	Age	Month of admission	Tmax (°C)	Cough	Dyspnea	Diarrhea	Length of onset to dyspnea (days)	Length of onset to ICU admission (days)	Ig G	IgM	PCR of sputum	PCR of BALF	PCR of pleural effusion	NGS from BALF	Combined Bacteria or virus	Length of ICU stay (days)	Length of hospital stay (days)
Case 1	Female	34	June	40.6	Dry cough	Yes	Yes	13	15	–	–	Positive	Positive	NA	Positive	Adenovirus	14	14
Case 2	Female	26	May	40.6	Dry cough	Yes	Yes	12	14	+	+	Positive	NA	NA	Positive	Rhinovirus Streptococcus	7	20
Case 3	Female	15	Dec.	40.5	Productive cough	Yes	Yes	15	15	+	+	Positive	NA	NA	NA	None	6	15
Case 4	Male	42	May	42	Dry cough	Yes	No	6	9	–	–	Positive	NA	NA	NA	Rhinovirus Epstein-Barr virus	12	12
Case 5	Male	32	Apr.	39.6	Dry cough	Yes	No	12	14	+	–	Positive	NA	NA	NA	Rhinovirus	13	13
Case 6	Male	44	Mar.	40	Dry cough	Yes	No	6	14	+	–	Positive	NA	NA	NA	Rhinovirus	3	13
Case 7	Male	53	Nov.	39.5	Productive cough	Yes	No	9	10	+	–	Positive	NA	NA	NA	None	4	17
Case 8	Female	17	Aug.	41.2	Dry cough	Yes	No	5	12	+	+	Positive	Positive	Positive	Positive	Acinetobacter baumannii	33	33
Case 9	Male	34	Oct..	39.6	Dry cough	Yes	No	9	11	+	+	Positive	Positive	NA	Positive	None	4	14
Case 10	Male	17	Nov	39.8	Dry cough	Yes	Yes	3	3	–	+	NA	NA	NA	Positive	Acinetobacter baumannii	9	18
Mean (±SD)	6/10 (60%)	31 (13)		40.4 (0.8)		10/10 (100%)	4/10 (40%)	9 (4)	11 (4)	+ (70%)	+ (50%)	9	3	1	5		11 (9)	17 (6.2)

*Tmax* Maximum temperature, *NGS* Next-generation sequencing detection, *BALF* Bronchoalveolar lavage fluid, *NA* Not available, *PCR* Polymerase chain reaction

### Microbiological findings

Seven (70%) and 5 (50%) patients had positive serum *M. pneumoniae* IgG and IgM, respectively. *M. pneumoniae* PCR of the sputum was performed in 9 (90%) cases, and was positive in all 9 cases. Three of the 9 cases had *M. pneumoniae* PCR from BALF at the same time, and all 3 cases (100%) were positive. Another patient was diagnosed with *M. pneumoniae* pneumonia through PCR of pleural effusion fluid. Five cases had mNGS from BALF, and all these 5 cases were positive for *M. pneumoniae* (Table 1).

*Acinetobacter baumannii* was detected in 2 patients (20%) who were transferred from another hospital after ICU admission, but these were isolated from the lower respiratory tract (LRT) samples collected after more than 3 days of their ICU stay, and therefore were not considered as causative agents of ARDS together with *M. pneumoniae* (Table 1).

### Clinical features of symptoms and signs, laboratory tests and radiologic findings

#### Symptoms and signs

All 10 patients had cough and fever at the onset of illness. They presented with a high fever, with a median body temperature of 40.3 °C (range, 39.6 °C to 42.0 °C). Eight patients (80%) had dry cough and two patients had productive cough. Four patients (40%) had diarrhea and one patient (10%) had abdominal pain (Table 1). Acute respiratory deterioration occurred 3 to 15 (median 9) days after the onset of symptoms. After the onset of dyspnea, patients usually progressed to acute respiratory failure. The mean time from symptom onset to ICU admission was 11 days (range, 3 to 15 days) (Table 1). All 10 cases had tachypnea when admitted to the ICU, with mean respiratory rate of 27 breaths per minute (range, 21 to 33).

#### Laboratory tests

Arterial blood gas analysis at ICU admission of all patients revealed hypoxia, with a mean PaO_2_/FiO_2_ of 180 (range = 47 to 263) mmHg. White blood cell counts were low or in the normal range on the first day of admission. All patients had elevated serum aspartate aminotransferase (AST), lactate dehydrogenase (LDH) and hydroxybutyrate dehydrogenase (HBDH). Eight patients (80%) had elevated AST (44 U/L to 134 U/L). Four patients (40%) had elevated CK (457 U/L to 1052 U/L). Nine patients (90%) had elevated LDH and HBDH (328 U/L to 920 U/L, and 196 U/L to 637 U/L) (Attached file 1, E-Table 1). Nine patients were tested for cell-mediated immunity, immunoglobulins (serum IgG, IgA and IgM), and components (Attached file 2, E-Table 2).

#### Characteristics of pleural effusions

In our study, eight patients had unilateral pleural effusions, and only one patient had bilateral pleural effusions. Pleural effusion fluid was examined in 4 patients. The pleural effusion fluid was light yellow and clear in 2 patients, and were yellow but turbid in the other 2 patients. Routine pleural effusion tests demonstrated mononuclear cell dominant leukocyte populations (Attached file 3, E-Table 3).

#### Radiologic findings

**Fig. 1:**
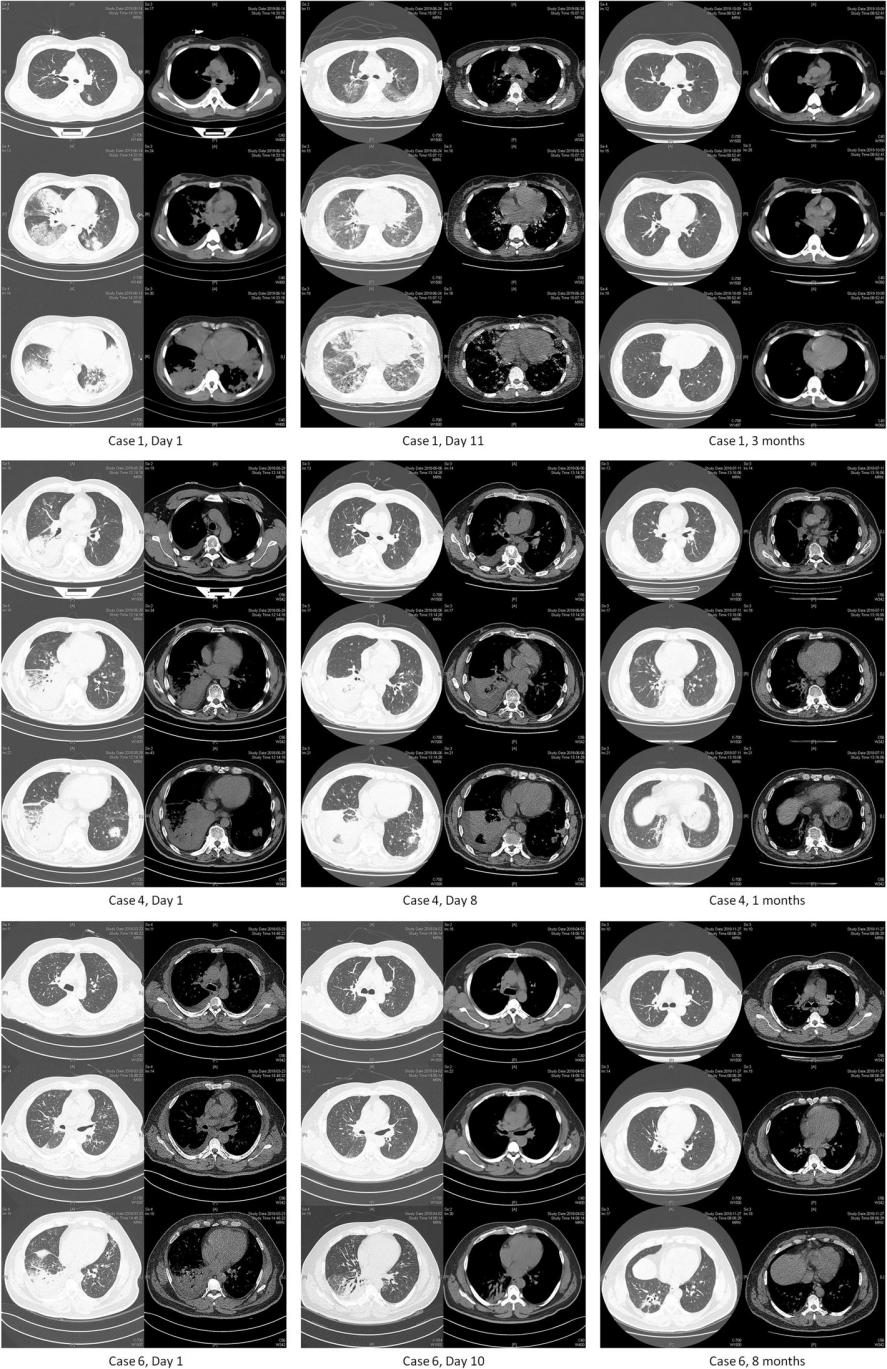
Chest computed tomography (CT) on day 1, day 8–11, followed up after 1 months to 8 months of three patients (case1, case 4 and case 6). Three representative slices of the upper, middle and lower lobe were chosen

All 10 patients had CXRs. CXRs revealed bilateral multi-lobular or segmental consolidation in nine (90%) patients. One patient’s CXR showed diffuse peribronchial infiltration. All 10 patients underwent chest HRCT. Unilateral or bilateral consolidation and infiltration were found on HRCT scans of 9 patients (90%). Large areas of consolidation within a single lobe or several lobes (90%), followed by pleural effusion (80%), were the most common findings on HRCT (Fig. 1). Only the HRCT of case 9 showed peribronchial infiltration without consolidation and pleural effusion.

### Respiratory mechanics and respiratory support

The average PaO_2_/FiO_2_ of all patients was 180 mmHg. Four (40%) cases had moderate ARDS (100 mmHg ≤ PaO_2_/FiO_2_ < 200 mmHg), and three cases (30%) had severe ARDS (PaO_2_/FiO_2_ < 100 mmHg). HFNC was applied in all patients (100%), with a median gas flow of 50 L/min (45-53 L/min) and FiO_2_ 0.48(0.35–0.5), but only two patients were sufficiently supported with HFNC. NIV was used in four patients with a median duration of 24(13–32) h, and one patient had NIV failure and needed intubation (Table 2).

**Table 2:** The setting of respiratory support and the characteristics of respiratory mechanics

Case No	Breathing rate (breaths/min)	PaO_2_/FiO_2_	HFNC (hours)	Setting of HFNC	NIV (hours)	Setting of NIV	IMV (days)	The mode and the initial parameter setting of IMV	SPO_2_ before RM	SPO_2_ after RM	Prone position	ECMO (days)	Resistance (L/cm H_2_O/S)	Compliance (ml/cm H_2_O)
Case 1	27	198	63	Oxygen flow 40 L/Min FiO_2_ 0.60	11	CPAP 5cmH_2_O FiO_2_ 0.7	10	P-A/C, Pi 12 cmH_2_O PEEPmax 16 cmH_2_O, FiO_2_ 1.0	73%	97%	Yes	0	20	34
Case 2	25	162	85	Oxygen flow 50 L/Min FiO_2_ 0.50	33	S/T 10/5cmH_2_O FiO_2_ 0.45	–	NA	NA	NA	NO	0	NA	NA
Case 3	21	118	20	Oxygen flow 60 L/Min FiO_2_ 0.50	–	NA	3	P-A/C, Pi 12 cmH_2_O PEEPmax 16 cmH_2_O, FiO_2_ 1.0	90%	98%	Yes	0	14	41
Case 4	33	155	158	Oxygen flow 50 L/Min FiO_2_ 0.45	–	NA	–	NA	NA	NA	Yes	0	NA	NA
Case 5	24	56	43	Oxygen flow 60 L/Min FiO_2_ 0.45	–	NA	8	P-A/C, Pi 10 cmH_2_O PEEPmax 14 cmH_2_O, FiO_2_ 1.0	92%	99%	Yes	0	15	54
Case 6	29	230	35	Oxygen flow 45 L/Min FiO_2_ 0.35	–	NA	–	NA	NA	NA	NA	0	NA	NA
Case 7	24	263	39	Oxygen flow 45 L/Min FiO_2_ 0.35	20	CPAP 6cmH_2_O FiO_2_ 0.28	–	NA	NA	NA	NA	0	NA	N A
Case 8	30	47	17	Oxygen flow 45 L/Min FiO_2_ 0.26	–	NA	15	P-A/C, Pi 10 cmH_2_O PEEPmax10 cmH_2_O, FiO_2_ 1.0	94%	94%	Yes	6	14	22
Case 9	27	260	54	Oxygen flow 50 L/Min FiO_2_ 0.5	28	S/T 10/6cmH_2_O FiO_2_ 0.5	–	NA	NA	NA	NA	0	NA	NA
Case 10	30	96	30	Oxygen flow 50 L/Min FiO_2_ 0.5	–	NA	7	P-A/C, Pi 10 cmH_2_O PEEPmax12 cmH_2_O, FiO_2_ 1.0	NA	NA	No	5	NA	NA
Total/median (IQR)	27 (21–33)	180 (47–263)	41 (28–69)	50 (45–53)L/min 0.48 (0.35–0.5)	24 (13–32)		8 (5–13)	PEEPmax 14 cm H_2_O (11–16)	91% (77–94%)	98% (95–99%)	YES 4 (40%)	5.5 (5–6)	15 (14–19)	38 (25–51)

*NIV* Non-invasive ventilation, *HFNC* High-flow nasal cannula, *IMV* Invasive mechanical ventilation, *ECMO* Extracorporeal membrane oxygenation support

Invasive mechanical ventilation (IMV) was carried out in 5 patients (50%). High airway resistance (median 15 L/cmH_2_O/s) and low respiratory system compliance (median 38 ml/cmH_2_O) was observed in all 5 cases. Recruitment maneuver (RM) was applied in 4 cases, with 1 patient not responsive to RM, and 4 cases were put into prone position. The maximum PEEP was 14 cmH_2_O (range, 11–16 cmH_2_O). Two cases (20%) needed ECMO support with median support duration of 5.5 days (Table 2). As shown in Table 2, Case 8 had the worst compliance and was unresponsive to RM, and ECMO was established. Case 1, case 3, and case 5 were responsive to RM and prone position.

### Treatment

All 10 patients did not receive fluoroquinolones at the onset of their illness, and switched to fluoroquinolones 8.7 ± 3.5 days after onset. All patients received β-lactams treatment as first therapy, and 5 patients received treatment combined with macrolides before their admission to ICU. After ICU admission, moxifloxacin were given to all 10 patients.

No patients in our case series received corticosteroid therapy.

### Outcomes

All patients survived in the ICU and were discharged from the hospital. The mortality of our cases was 0%. The average length of ICU stay was 11 days, and the average length of hospital stay was 17 days.

### Comparison of intubated and non-intubated cases in MPP patients

As shown in Table 3, compared to non-intubated patients, patients in intubated group were younger, were less likely to be male, had lower PaO_2_/FiO_2_ and higher APACHE II scores, and had higher procalcitonin and neutrophil proportion at ICU admission.

**Table 3:** Comparisons between patients with IMV and non-IMV (Median, IQR)

	Non-intubated group *n* = 5	IMV group *n* = 5	*P* value
Male(n,%)	4 (80%)	2 (40%)	0.003*
Age (year)	42 (30–49)	17 (16–33)	0.032*
APACHEII	6 (4–12)	17 (11–21)	0.028*
PaO_2_/FiO_2_	230 (159–262)	96 (51–158)	0.032*
Lactate	0.9 (0.7–1.3)	1.3 (1.2–1.7)	0.056
Breathing rate (breaths/min)	26 (25–31)	27 (23–29)	0.690
Tmax((°C))	40 (39.5–41.3)	40 (39.7–40.9)	0.841
Procalcitonin	0.175 (0.14–0.195)	1.05 (0.45–2.09)	0.016*
White blood cell count(10^9^/L)	4.5 (3.8–7.3)	7.6 (5.5–8.7)	0.151
Neutrophil proportion(%)	73 (54–82)	92 (83–94)	0.016*
AST (U/L)	81 (33–102)	100 (41–121)	0.548
Albumin (g/L)	29 (28–34)	32 (26–39)	1.0
Pre-albumin (g/L)	0.08 (0.02–0.15)	0.03 (0.03–0.04)	0.571
Creatine (umol/L)	55 (47–69)	43 (35–76)	1.0
C-reactive protein (CRP, mg/L)	17 (6–48)	93 (11–209)	0.286
Erythrocyte sedimentation rate (ESR,mm/h)	25 (5.5–28.5)	12 (4–25)	0.675
Length of onset to ICU admission (days)	11 (9.5–14)	14 (7.5–15)	0.421
Length of ICU stay (days)	4 (3–18.5)	13 (17.5–23.5)	0.056

*APACHE II* Acute physiological and chronic health evaluation II score; *P < 0.05

## Discussion

To our knowledge, our study is the first and largest case series to evaluate diagnostic and therapeutic modalities in severe *M. pneumoniae* pneumonia induced ARDS. Our main findings are as follows: 1) early and rapid diagnosis for severe *M. pneumoniae* pneumonia with ARDS was achieved with PCR/mNGS test of samples from the lower respiratory tract and pleural effusions; 2) CT findings mainly showed alveolar patterns with bilateral consolidations rather than interstitial patterns; 3) respiratory mechanics showed low respiratory system compliance and high airway resistance; 4) 50% of *M. pneumoniae* induced ARDS were adequately supported with HFNC or NIV, 50% required intubation, RM and prone position were effective in 30% intubated cases, and 20% needed ECMO support; 5) when early anti-mycoplasmal drugs together with sufficient respiratory support are given, the survival rate was high with no need for corticosteroids; and 6) younger patients with lower PaO_2_/FiO_2_ and APACHE II scores, and higher PCT and higher neutrophil cell proportion at ICU admission were more likely to require intubation.

In our study, the clinical manifestations of severe *M. pneumoniae* pneumonia induced ARDS were primarily dry cough, high fever, and acute respiratory failure with bilateral consolidations on radiologic examination. Respiratory failure occurred a median of 9 days (range, 3 to 15 days) after onset of symptoms, similar to the previous descriptions [3, 29]. However, these clinical features are not specific for early recognition and diagnosis of severe *M. pneumoniae* pneumonia. Therefore, early and precise laboratory detection of *M. pneumoniae* infection is essential to prevent deterioration. Previous methods, such as mycoplasma culture and serological tests, which may require several weeks, are not practical. As presented in our study, early definitive diagnosis is now dependent on PCR or mNGS [25], which had high specificity and sensitivity. Thus, further development of these relatively new diagnostic tools is warranted, and should be applied in cases of severe CAP induced ARDS with suspected *M. pneumoniae* infection.

Furthermore, our study found that most of our cases had pleural effusion, and PCR was positive for *M. pneumoniae* in pleural effusion fluid. Similar findings were also reported in a previous case report [24]. Therefore, in patients with dry cough and difficulty obtaining a lower respiratory sample, early PCR/mNGS for *M. pneumoniae* using pleural effusion fluid may be an option. As ARDS is a clinical syndrome with many different causes and may induced by some less common pathogens, mNGS is used in our ICU for early detection of possible unknown etiology, and we found that mNGS had a good value in diagnosis for *M. pneumoniae* in our cases. However, in most cases mNGS is more appropriate to be used for patients with unknown etiology, and may not be suitable for routine examination of some common pathogens such as ADV, RSV, and *M. pneumoniae*. Thus, once commercial PCR kits are available for diagnosis of *M. pneumoniae* infection, it is not necessary to detect *M. pneumoniae* using mNGS as a primary option.

In both our case series and previously reported cases of *M. pneumoniae* associated ARDS, deterioration of the clinical state presumably due to a significant period of inadequate antibiotic treatment [29, 30]. In a review of severe or fatal *M. pneumoniae* pneumonia, the average duration from onset of infection to the development of respiratory failure was 11.2 days (range, 5–21 days) [29]. Chan and Miyashita et al. reported durations of 10–15 and 9.3 days, respectively, from onset to first administration of appropriate anti-mycoplasma agents [4, 5]. The duration of an average of 9 days to change the treatment from our study was similar to the previous studies. Therefore, our management would still be considered as late intervention, and the delay as a risk factor for development of respiratory failure and ARDS. Earlier recognition of *M. pneumonia*e in the differential diagnosis and earlier initiation of appropriate antibiotics would potentially prevent *M. pneumoniae* pneumonia from progressing to ARDS.

Furthermore, more awareness is needed on the emergence of macrolide-resistant *M. pneumoniae* infection in adults [31, 32]. A previous report from our center found the rate of resistance to macrolides was 88.3% of the isolated *M. pneumoniae,* and all resistant strains harbored A2063G mutations. The isolated macrolide-resistant *M. pneumoniae* were resistant to erythromycin, and also showed cross-resistance to clarithromycin and azithromycin. All isolates were sensitive to tetracyclines and fluoroquinolones. Moxifloxacin was more active than ciprofloxacin and levofloxacin [33]. However, sequencing of macrolide resistance genes is not a routine test in clinical practice in our centers, and we did not perform sequencing for macrolide resistance genes in our cases. We speculated that we have the similar high rate of resistance and similar type of resistant genes in our case series. Thus, early fluoroquinolones were considered as first line treatment for *M. pneumonia* induced severe ARDS cases in adults.

A previous epidemiological study from one of our centers (Beijing Chao-Yang Hospital) that routinely screened for *M. pneumoniae* in outpatients during 2011–2016 determined that only 14 patients out of 1127 patients (1.2%) with *M. pneumoniae* infection needed ICU admission [21]. However, after that study concluded, *M. pneumoniae* infection was only routinely screened in patients with a diagnosis of community acquired pneumonia who were hospitalized in our general ward or admitted to our ICU. During our study period, 11 of 418 severe CAP were admitted to our ICU and were diagnosed with *M. pneumoniae* pneumonia (2.3%). Additionally, as shown in Table 1 in our study, the first LRT sample for *M. pneumoniae* was collected on an average of 11 ± 4 days after the onset of symptoms. The higher rate of *M. pneumoniae* pneumonia in our case series suggests that early detection for the pathogen may be needed to start an early intervention and proper treatment.

The 3 patients with mild ARDS in our study were successfully supported by HFNC and NIV without intubation. One patient with moderate ARDS was successfully supported with a combination of HFNC and awake prone positioning, which proved safe and effective in moderate ARDS patients by our team in a prospective study [34]. HFNC or NIV, combined with early prone positioning, may be a new support strategy for acute respiratory failure in *M. pneumoniae* indunced mild to moderate ARDS patients.

Although the radiologic findings showed a diffuse alveolar pattern with consolidations and the respiratory mechanics showed decreased respiratory system compliance, most intubated patients were responsive to RM and prone positioning during invasive ventilation, with a maximum PEEP of 11–16 (median 14) cmH_2_O was applied. However, two cases deteriorated to severe hypoxia despite anti-mycoplasmal therapy and invasive ventilation, eventually requiring ECMO support. In a recent case report and literature review for use of ECMO in *M. pneumoniae* associated ARDS, the mean ECMO run was 232 h/9.68 days [28], similar to that of our cases. The overall survival rate for 22 cases of *M. pneumoniae* requiring ECMO with reported outcome was 72.7%(16/22), demonstrating that ECMO may be safely and effectively used to treat severe ARDS caused by *M. pneumoniae* infection [28].

Previous reports support the hypothesis that the severity of the disease and pulmonary infiltrates may be directly correlated with the level of the individual immune response. However, in our study, we did not observe significant increases of cell or humoral immunity as demonstrated by T cell subset cell count or immunoglobulin levels in more severe disease. Most interestingly, we found that with appropriate respiratory support and anti-mycoplasmal therapy, all patients had a rapid clinical improvement. Therefore, no corticosteroids were given, and all patients finally recovered from ARDS without corticosteroid use. Prolonged or inappropriate use of corticosteroids may cause excess downregulation of cell-medicated immunity and result in immunosuppression, making individuals more susceptible to more severe *M. pneumoniae* infection or opportunistic infections. A recent case report revealed that *M. pneumoniae* associated ARDS had no elevated pulmonary vascular permeability, and was successfully treated using low-dose short-term hydrocortisone, suggesting that pulmonary infiltration in ARDS caused by *M. pneumoniae* does not match the criteria of permeability edema observed in typical ARDS [35]. Therefore, careful consideration is required when deciding whether to use high dose corticosteroid in the future cases similar to ours.

There are several limitations for our study. First, performing statistical analysis on a small sample size was prone to bias, potentially yielding spurious findings. Increasing the sample size and collecting more cases in a further study may avoid this kind of limitation. Second, this study is a retrospective study with the associated limitations on complete data collection.

## Conclusions

In conclusion, early and rapid diagnosis for severe *M. pneumoniae* pneumonia with ARDS can be achieved by PCR/mNGS test in samples from lower respiratory tract or pleural effusion. In our case series, half of *M. pneumoniae* induced ARDS cases were adequately supported with HFNC or NIV and 50% required intubation. RM and prone position were effective in 30% of intubated cases, and 20% needed ECMO support. When early anti-mycoplasmal therapy was given together with sufficient respiratory support, the survival rate was high with no need for corticosteroid use.

## Supplementary information

**Additional file 1. **E-Table 1. The laboratory findings for the patients with severe *M. Pneumoniae* pneumonia on the first day of admission.

**Additional file 2.** E-Table 2. The result of pleural effusion biochemistry and pleural effusion routine of the 4 severe *M. pneumoniae* pneumonia.

**Additional file 3.** E-Table 3. Cell-mediated immunity and Humoral immunity on the immunocompetent patients with severe *M. Pneumoniae* pneumonia on the first day of admission.

## Data Availability

The datasets used and/or analysed during the current study are available from the corresponding author on reasonable request.

## Abbreviations

ARDS: Acute respiratory distress syndrome
BALF: Broncheoalveolar lavage fluid
CXR: Chest X-ray
ECMO: Extracorporeal membrane oxygenation
HFNC: High-flow nasal cannula
HRCT: High-resolution computed tomography
ICU: Intensive care unit
IMV: Invasive mechanical ventilation
LRT: Lower respiratory tract
NIV: Non-invasive ventilation
