# Supplementary material for: Early diagnosis and appropriate respiratory support for Mycoplasma pneumoniae pneumonia associated acute respiratory distress syndrome in young and adult patients: a case series from two centers
Source: BMC Infect Dis. 2020 May 24;20:367. doi: 10.1186/s12879-020-05085-5 (PMC7245847; doi:10.1186/s12879-020-05085-5)
Supplement: Supplementary file 1 — Additional file 1. E-Table 1. The laboratory findings for the patients with severe M. Pneumoniae pneumonia on the first day of admission. [file 12879_2020_5085_MOESM1_ESM.docx]

E-Table 1. The laboratory findings for the patients with severe *M. Pneumoniae* pneumonia on the first day of admission

|  | WBC (*10^9^/L) | NE(%) | AST (U/L) | ALB (g/L) | PALB (g/L) | Crea (μmol/L) | CK (U/L) | LDH (U/L) | HBDH (U/L) |
| --- | --- | --- | --- | --- | --- | --- | --- | --- | --- |
| case1 | 9.19 | 93.1 | 107 | 33.4 | NA | 97 | 600 | 603 | 432 |
| case2 | 3.65 | 72.8 | 44 | 28.5 | 0.12 | 197 | 32 | 539 | 406 |
| case3 | 4.5 | 80.9 | 100 | 31.9 | 0.03 | 159 | 1052 | 767 | 530 |
| case4 | 3.89 | 54.5 | 81 | 29.2 | 0.03 | 137 | 645 | 592 | 435 |
| case5 | 7.61 | 86 | 134 | 25.4 | 0.04 | 198 | 457 | 920 | 637 |
| case6 | 5.47 | 54 | 117 | 28.9 | 0.17 | 104 | 169 | 409 | 330 |
| case7 | 4.5 | 84.4 | 86 | 27.7 | 0.01 | 93 | 143 | 258 | 196 |
| case8 | 8.18 | 92.3 | 54 | 27.5 | 0.03 | 230 | 155 | 525 | 406 |
| case9 | 9.03 | 79.5 | 22 | 39.2 | 0.08 | 157 | 185 | 245 | 209 |
| case10 | 6.49 | 94.8 | 27 | 43.6 | NA | 168 | 153 | 328 | NA |
| Mean (±SD) | 5.94 (2.02) | 77.1 (17.0) | 77.2 (38.8) | 31.5 (5.8) | 0.06 (0.05) | 154 (47) | 359 (322) | 519 (218) | 398 (130) |

WBC, White blood cell count (normal range = 4.00 to 10.00*10^9^/L). NE, Neutrophil.AST, Aspartate aminotransferase (normal range = 10 to 42 U/L). CK, Creatine phosphokinase (normal range = 38 to 174 U/L). HBDH, Hydroxybutyrate dehydrogenase.(normal range = 72 to 182 U/L). LDH, Lactate dehydrogenase (normal range = 85 to 250 U/L). WBC, White blood cell count (normal range = 4.00 to 10.00*10^9^/L). NA, not available
