# Supplementary material for: Early diagnosis and appropriate respiratory support for Mycoplasma pneumoniae pneumonia associated acute respiratory distress syndrome in young and adult patients: a case series from two centers
Source: BMC Infect Dis. 2020 May 24;20:367. doi: 10.1186/s12879-020-05085-5 (PMC7245847; doi:10.1186/s12879-020-05085-5)
Supplement: Supplementary file 2 — Additional file 2. E-Table 2. The result of pleural effusion biochemistry and pleural effusion routine of the 4 severe M. pneumoniae pneumonia. [file 12879_2020_5085_MOESM2_ESM.docx]

E-Table 2. The result of pleural effusion biochemistry and pleural effusion routine of the 4 severe M. pneumonae pneumonia.

|  | Outlook | Rivalta | ADA  (u/L） | LDH  (u/L) | Total protein  (g/L) | CL  (mmol/L) | Glucose  (mmol/L) | The count of all cells  (/um) | WBC count  (/um) | Mononuclear cells (%) | Neutrophil cells (%) | PCR of pleural effusion |
| --- | --- | --- | --- | --- | --- | --- | --- | --- | --- | --- | --- | --- |
| Case1 | Light yellow | + | 11 |  | 20.6 | 113.3 | 7.35 | 1163 | 163 | 80 | 20 | NA |
| Case3 | Light yelow | _ | 16 | 593 | 42.6 | 107.8 | 5.96 | 2291 | 291 | 90 | 10 | NA |
| Case5 | Yellow  and turbid | ++ | 25 | 2322 | 29.7 | 111.3 | 5.15 | 6413 | 413 | 85 | 15 | NA |
| Case8 | Yellow  and  turbid | _ | 29 | 1224 | 32 | 114 | 6.59 | 10724 | 504 | 70 | 30 | Mycoplasma pneumoniae |
| Median  (IQR) |  |  | 20.5  (12.3-28) | 1224  (593-2322) | 31  (23-40) | 112  (109-114) | 6.28  (5.4-7.2) | 4352  (1445-9646) | 352  (195-481) | 83  (73-89) | 17(11-28) |  |

ADA, adenosine deaminase . WBC, white blood cell. LDH, lactic dehydrogenase. Cl, chlorine. PCR, polymerase chain reaction.
