# Supplementary material for: Early diagnosis and appropriate respiratory support for Mycoplasma pneumoniae pneumonia associated acute respiratory distress syndrome in young and adult patients: a case series from two centers
Source: BMC Infect Dis. 2020 May 24;20:367. doi: 10.1186/s12879-020-05085-5 (PMC7245847; doi:10.1186/s12879-020-05085-5)
Supplement: Supplementary file 3 — Additional file 3. E-Table 3. Cell-mediated immunity and Humoral immunity on the immunocompetent patients with severe M. Pneumoniae pneumonia on the first day of admission. [file 12879_2020_5085_MOESM3_ESM.docx]

E-Table 3 Cell-mediated immunity and Humoral immunity on the immunocompetent patients with severe MPP on the first day of admission

| Patient | T cells (n/ul) | T4 cells (n/ul) | T8-cells (n/ul) | IgG (mg/dl) | IgA (mg/dl) | IgM (mg/dl) | C3 (mg/dl) | C4 (mg/dl) |
| --- | --- | --- | --- | --- | --- | --- | --- | --- |
| Reference | (770 to 2,040) | (410 to 1,120) | (240 to 880) | (751 to 1,560) | (82 to 453) | (46 to 304) | (79 to 152) | (12 to 36) |
| 1 | 410 | 227 | 162 | 646.0 | 173.0 | 125.0 | 92.3 | 27.9 |
| 2 | 512 | 315 | 191 | 836.0 | 135.0 | 232.0 | 64.5 | 7.46 |
| 3 | 1704 | 589 | 1166 | 981.0 | 95.5 | 343.0 | 81.8 | 19.4 |
| 4 | 987 | 695 | 248 | 825.0 | 291.0 | 126.0 | 106.0 | 37.0 |
| 5 | 317 | 203 | 166 | 478.0 | 92.5 | 146.0 | 55.7 | 20.3 |
| 6 | 919 | 605 | 289 | 1110.0 | 235.0 | 71.0 | 78.6 | 21.3 |
| 7 | 508 | 344 | 157 | 959.0 | 217.0 | 36.3 | 63.7 | 23.4 |
| 8 | 474 | 277 | 197 | 940.0 | 112.0 | 181.0 | 21.1 | 4.43 |
| 9 | 699 | 379 | 211 | 1020 | 265 | 161 | 88.5 | 15.8 |
| Mean  (±SD) | 726  (431) | 407  (192) | 309  (323) | 866.1  (197.5) | 179.6  (75.4) | 157.9  (90.1) | 72.5  (24.9) | 19.7  (9.9) |
